# Case Report of Leprosy in Central Florida, USA, 2022

**DOI:** 10.3201/eid2908.220367

**Published:** 2023-08

**Authors:** Aashni Bhukhan, Charles Dunn, Rajiv Nathoo

**Affiliations:** Kansas City University–Graduate Medical Education/Advanced Dermatology and Cosmetic Surgery Consortium, Orlando, Florida, USA

**Keywords:** Leprosy, Florida, Hansen disease, lepromatous leprosy, *Mycobacterium leprae*, armadillos, bacteria, tuberculosis and other mycobacteria, United States

## Abstract

Florida, USA, has witnessed an increased incidence of leprosy cases lacking traditional risk factors. Those trends, in addition to decreasing diagnoses in foreign-born persons, contribute to rising evidence that leprosy has become endemic in the southeastern United States. Travel to Florida should be considered when conducting leprosy contact tracing in any state.

Leprosy, or Hansen disease, is a chronic infectious disease caused by the acid-fast rod *Mycobacterium leprae.* Leprosy primarily affects the skin and peripheral nervous system, and disease course is largely dependent on individual susceptibility to *M. leprae* (*1*). Leprosy has been historically uncommon in the United States; incidence peaked around 1983, and a drastic reduction in the annual number of documented cases occurred from the 1980s through 2000 (*2*). However, since then, reports demonstrate a gradual increase in the incidence of leprosy in the United States. The number of reported cases has more than doubled in the southeastern states over the last decade (*2*). According to the National Hansen’s Disease Program, 159 new cases were reported in the United States in 2020; Florida was among the top reporting states (*2*).

Central Florida, in particular, accounted for 81% of cases reported in Florida and almost one fifth of nationally reported cases (*3*). Whereas leprosy in the United States previously affected persons who had immigrated from leprosy-endemic areas, ≈34% of new case-patients during 2015–2020 appeared to have locally acquired the disease (*4*). Several cases in central Florida demonstrate no clear evidence of zoonotic exposure or traditionally known risk factors. We report a case of lepromatous leprosy in central Florida in a man without risk factors for known transmission routes. We also review the mounting epidemiologic evidence supporting leprosy as an endemic process in the southeastern United States.

A 54-year-old man sought treatment at a dermatology clinic for a painful and progressive erythematous rash (Figure). The lesions began on his distal extensor extremities and progressed to involve his trunk and face. He denied any domestic or foreign travel, exposure to armadillos, prolonged contact with immigrants from leprosy-endemic countries, or connections with someone known to have leprosy. He has resided in central Florida his entire life, works in landscaping, and spends long periods of time outdoors. Biopsies of multiple sites demonstrated a diffuse dermal infiltrate composed of disorganized aggregates of foamy histiocytes and lymphocytes. Fite stains revealed acid-fast bacilli within histiocytes and cutaneous nerve twigs, a pathognomonic finding of leprosy. He was referred to an infectious disease specialist who, under the direction of the National Hansen’s Disease Program, prescribed triple therapy with dapsone, rifampin, and clofazimine.

**Figure:**
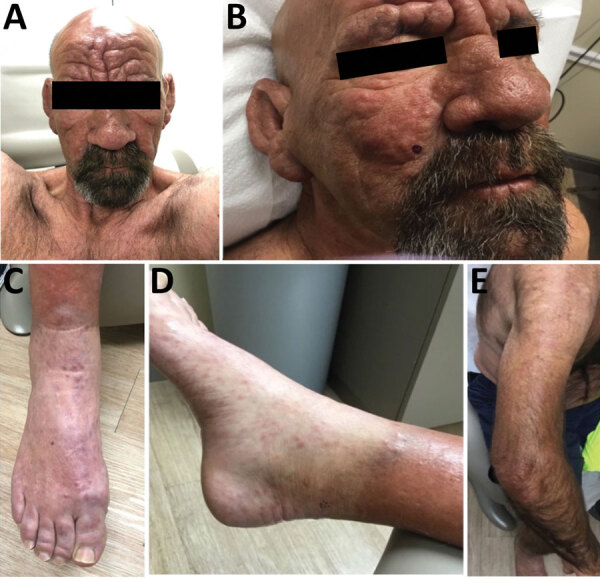
Lepromatous leprosy in a 54-year-old man in central Florida, USA, 2022. A, B) Leonine facies with waxy yellow papules. C) Violaceous nonblanching macules coalescing into patches along dorsum of feet bilaterally. D, E) Erythematous papules coalescing into plaques along extensor aspects of upper and lower extremities bilaterally. Plaques notably demonstrated a moderate degree of dysesthesia.

Transmission of leprosy has not been fully elucidated. Prolonged person-to-person contact through respiratory droplets is the most widely recognized route of transmission (*1*). A high percentage of unrelated leprosy cases in the southern United States were found to carry the same unique strain of *M. leprae* as nine-banded armadillos in the region, suggesting a strong likelihood of zoonotic transmission (*4*). A recent systematic review analyzing studies conducted during 1945–2019 supports an increasing role of anthroponotic and zoonotic transmission of leprosy (*5*). However, Rendini et al. demonstrated that many cases reported in eastern United States, including Georgia and central Florida, lacked zoonotic exposure or recent residence outside of the United States (*6*).

Given those reports, there is some support for the theory that international migration of persons with leprosy is a potential source of autochthonous transmission. Reports from Spain linked an increase in migration from other countries to an increase in autochthonous leprosy (*7*). The number of international migrants in North America increased from 27.6 million persons in 1990 to 58.7 million in 2020 (*8*), so a link to migration may account for the increase in incidence of leprosy in historically nonendemic areas. Further, reports from the Centers for Disease Control and Prevention show that, although the incidence of leprosy has been increasing, the rates of new diagnoses in persons born outside of the United States has been declining since 2002 (Appendix Figure) (*9*). This information suggests that leprosy has become an endemic disease process in Florida, warranting further research into other methods of autochthonous transmission.

Leprosy is a reportable condition in the state of Florida and is monitored primarily through passive surveillance. According to the Florida Department of Health, practitioners are required to report leprosy in Florida by the next business day (*10*). Contact tracing is critical to identifying sources and reducing transmission. In our case, contact tracing was done by the National Hansen’s Disease Program and revealed no associated risk factors, including travel, zoonotic exposure, occupational association, or personal contacts. The absence of traditional risk factors in many recent cases of leprosy in Florida, coupled with the high proportion of residents, like our patient, who spend a great deal of time outdoors, supports the investigation into environmental reservoirs as a potential source of transmission.

In summary, our case adds to the growing body of literature suggesting that central Florida represents an endemic location for leprosy. Travel to this area, even in the absence of other risk factors, should prompt consideration of leprosy in the appropriate clinical context. By increasing local physician efforts to report incidence and supporting further research to assess routes of transmission, a congruent effort can be made to identify and reduce spread of the disease.

Appendix Additional information for a case report of leprosy in central Florida, USA, 2022.
