# Supplementary material for: Case Report of Leprosy in Central Florida, USA, 2022
Source: Emerg Infect Dis. 2023 Aug;29(8):1698–700. doi: 10.3201/eid2908.220367 (PMC10370849; doi:10.3201/eid2908.220367)
Supplement: Appendix — Additional information for a case report of leprosy in central Florida, USA, 2022. [file 22-0367-Techapp-s1.pdf]

Article DOI: http://doi.org/10.3201/eid2908.220367

EID cannot ensure accessibility for supplementary materials supplied by authors. Readers who have difficulty accessing supplementary content should contact the authors for assistance.

Case Report of Leprosy in Central Florida, USA, 2022

Appendix

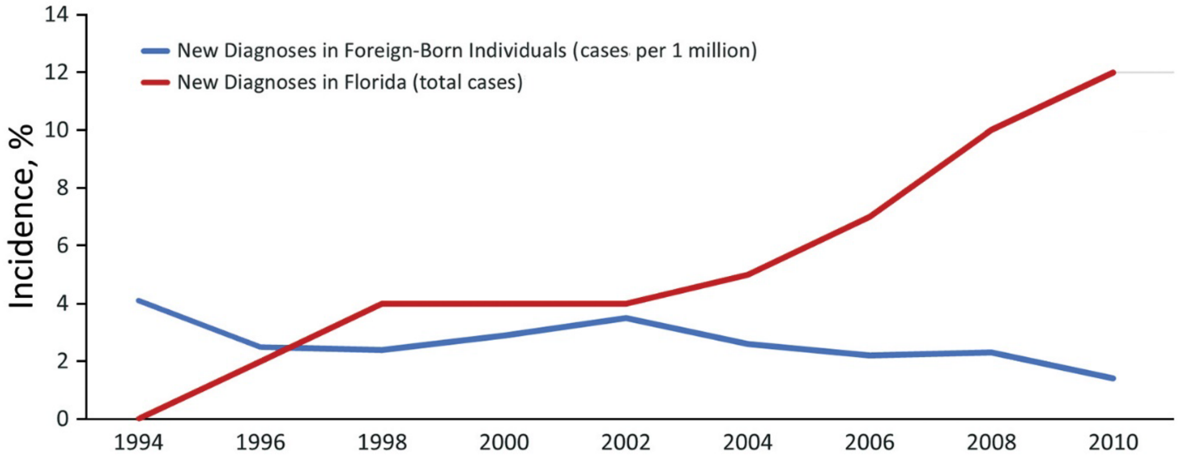

Appendix Figure. Decreasing number of new diagnoses of leprosy in foreign-born individuals in the United States as the overall incidence increases in Florida during 1994–2010.
